# Protocol for Northern Ireland Caries Prevention in Practice Trial (NIC-PIP) trial: a randomised controlled trial to measure the effects and costs of a dental caries prevention regime for young children attending primary care dental services

**DOI:** 10.1186/1472-6831-11-27

**Published:** 2011-10-10

**Authors:** Martin Tickle, Keith M Milsom, Michael Donaldson, Seamus Killough, Ciaran O'Neill, Grainne Crealey, Matthew Sutton, Solveig Noble, Margaret Greer, Helen V Worthington

**Affiliations:** 1 School of Dentistry, the University of Manchester, Oxford Road, Manchester, M13 9PL, UK; 2 Health and Social Care Board, County Hall. 182 Galgorm Road, Ballymena, County Antrim, Northern Ireland, BT42 1QB, UK; 3 British Dental Association, 2 Woodstock Link, Belfast, County Antrim, Northern Ireland, BT6 8DD, UK; 4 Department of Economics, National University of Ireland, University Road Galway, County Galway, Ireland; 5 Northern Ireland Clinical Research Support Centre, Education and Research Centre, Royal Group of Hospitals Trust, Grosvenor Road, Belfast, Northern Ireland, BT12 6BA, UK; 6 School of Community Based Medicine, The University of Manchester, Oxford Road, Manchester, M13 9PL, UK; 7 Northern Health and Social Care Trust, Greenmount Avenue, Ballymena, County Antrim, Northern Ireland, BT43 6DA, UK

## Abstract

**Background:**

Dental caries is a persistent public health problem with little change in the prevalence in young children over the last 20 years. Once a child contracts the disease it has a significant impact on their quality of life. There is good evidence from Cochrane reviews including trials that fluoride varnish and regular use of fluoride toothpaste can prevent caries.

The Northern Ireland Caries Prevention in Practice Trial (NIC-PIP) trial will compare the costs and effects of a caries preventive package (fluoride varnish, toothpaste, toothbrush and standardised dental health education) with dental health education alone in young children.

**Methods/Design:**

A randomised controlled trial on children initially aged 2 and 3 years old who are regular attenders at the primary dental care services in Northern Ireland. Children will be recruited and randomised in dental practices. Children will be randomised to the prevention package of both fluoride varnish (twice per year for three years), fluoride toothpaste (1,450 ppm F) (supplied twice per year), a toothbrush (supplied twice a year) or not; both test and control groups receive standardised dental health education delivered by the dentist twice per year. Randomisation will be conducted by the Belfast Trust Clinical Research Support Centre ([CRSC] a Clinical Trials Unit).

1200 participants will be recruited from approximately 40 dental practices. Children will be examined for caries by independent dental examiners at baseline and will be excluded if they have caries. The independent dental examiners will examine the children again at 3 years blinded to study group.

The primary end-point is whether the child develops caries (cavitation into dentine) or not over the three years. One secondary outcome is the number of carious surfaces in the primary dentition in children who experience caries. Other secondary outcomes are episodes of pain, extraction of primary teeth, other adverse events and costs which will be obtained from parental questionnaires.

**Discussion:**

This is a pragmatic trial conducted in general dental practice. It tests a composite caries prevention intervention, which represents an evidence based approach advocated by current guidance from the English Department of Health which is feasible to deliver to all low risk (caries free) children in general dental practice. The trial will provide valuable information to policy makers and clinicians on the costs and effects of caries prevention delivered to young children in general dental practice.

**Trial registration:**

EudraCT No: 2009 - 010725 - 39

ISRCTN: ISRCTN36180119

Ethics Reference No: 09/H1008/93:

## Background

Although dental caries is a preventable disease it is a persistent public health problem, with little change in the prevalence in young children over the last 20 years [1]. Caries is closely associated with social deprivation resulting in large geographical and social inequalities across the UK. The last national child dental health survey [1] showed that in 2003 43% and 61% of 5-year-olds in England and Northern Ireland respectively had caries; 10 years earlier, the figures were 45% and 60%. Over the last 10 years the proportion of parents taking children under 3 years to the dentist increased from 42% to 54% [1]; evidently this favourable change in visiting patterns has not been translated into reduced levels of tooth decay in the population.

Few prospective studies have been undertaken to provide an understanding of how the disease behaves longitudinally [2] A recently completed prospective cohort study [2,3] followed 739 children aged 3 to 6 years attending 50 dental practices in the North West of England over a 3 year period. This study demonstrated a stark difference between children who present with and without the disease at their first visit to the dentist. Over the study period 25% of caries free children developed caries, by contrast 72% of those with the disease at initial presentation developed further cavities. No matter what age a child contracted the disease it progressed at the same rapid rate. An important finding of this study was that more cases (children with caries) arose from the initially caries free population (N = 155, 21% of the total population and 25% of the population who were caries free at first attendance) than from those who present with the disease at their first visit to the dentist (N = 118, 16%). NHS Business Services Organisation (BSO) data shows that this situation is mirrored in Northern Ireland; 25% of 2-3-year-old children have the disease at initial presentation but 35% who are caries free at their first visit go on to develop the disease over a 3 year period. Once a child contracts the disease there is a significant impact on their quality of life and that of their family. Children with caries have an 18.8% chance each year of an unscheduled visit due to toothache and an 11% chance of an extraction each year [4]. As adverse outcomes are so common in children with the disease, the priority should be prevention, with a primary focus of maintaining the caries free children in that state. Dentists cannot prevent the disease starting in children who already have caries at their first visit; these children should be considered as a separate population; their dental care needs are quite different and are complicated by the effects of restorative treatment.

The need to improve preventive care provided by dentists has moved up the policy agenda following the publication of the Primary Dental Care Strategy for Northern Ireland [5] in 2006, which placed a strong emphasis on prevention of caries in general practice, and the subsequent Oral Health Strategy for Northern Ireland in 2007 [6], which sets targets for reduction in the caries levels of 5-year-olds. The introduction of new, locally commissioned NHS dental contracts in England and Wales in April 2006 also means that strengthening the evidence base for prevention is important for policy makers and the NHS. One of the main reasons for changing NHS dental contracts was to encourage prevention, however the new contract in England has been heavily criticised by the dental profession and NHS managers [7] and more recently by the House of Commons Health Select Committee [8] for offering little incentive for dentists to provide preventive care. Indeed one of the recommendations of the Health Select Committee report was that 'the Department of Health undertake research to determine the extent to which the provision of preventive advice is being given and its cost-effectiveness.' This emphasis on prevention is also seen in the Darzi report [9] and the Primary and Community Care Strategy [10]. Scotland has decided to retain the centrally funded GDS contract based on capitation for children and fee for item for adults. The Department of Health in Northern Ireland intends to change the GDS contract and wants to use the outcomes of this trial to inform preventive aspects of the new contract. So, there is pressure on politicians, policy makers and NHS commissioners in the UK to ensure that effective caries prevention is provided in practice. Unfortunately recent research suggests that the preventive care currently provided by GDPs is ineffective and inequitable [11]. Dentists are ill-equipped in terms of their knowledge [12] and how they present information to their patients [13] to provide an effective service.

Preventive care provided by most dentists is based on health education aimed at reducing sugar intake [12,13] which lacks evidence to demonstrate its effectiveness [14,15]. However, there is good evidence that fluoride-based interventions can have a dramatic effect on the disease. For example in a systematic review of water fluoridation [16] the median of mean differences of studies suggested that a 15% absolute difference in the proportions of children caries free can be expected between fluoridated and non-fluoridated populations. It has been estimated that this equates to a difference of around 40% in caries increment [17]. In England water fluoridation is currently being examined as a means of preventing caries but is not technically, economically or politically feasible in every area of the UK, so other delivery vehicles such as professionally applied fluoride varnish or distributed fluoride toothpaste need to be considered. A Cochrane systematic review of fluoride varnish [18] included 9 RCTs and reported a pooled d(e/m)fs prevented fraction estimate of 33% (95% CI, 19% to 48%; p < 0.0001). A second systematic review [19] of fluoride varnish used different selection criteria and identified only 3 trials examining primary teeth and concluded that the evidence was inconclusive due to the poor quality of the studies. A subsequently published trial of 2-4 year old children [20] examined the effect of 22,600 ppm varnish applied twice a year over 2 years and reported a 57% reduction in caries increment compared to a control group. Another recent trial in the USA [21] investigated the use of 22,600 ppm varnish on infants with a mean age of 1.8 years resident in an area supplied with artificially fluoridated water at 1 ppm. Caries incidence was lower in those receiving fluoride varnish twice a year than in a counselling only control (OR 3.77 (95% CI 1.88-7.58) and no adverse events were reported.

A Cochrane review of fluoride toothpaste use [22] in children aged 5-16 years reported clear evidence that fluoride toothpastes are efficacious in preventing caries in permanent teeth but there was little information concerning the primary dentition or adverse effects. Similar findings were also reported by another systematic review of toothpastes published around the same time[23]. An RCT in the North West [24] testing fluoride toothpaste provided through the post to children from birth to 5-years-old reported a 16% difference in increment and an 8% absolute difference in the proportion of caries free children receiving 1450 ppm toothpaste compared to a control group. A Cochrane systematic review examined the effectiveness of any fluoride agent (gel, varnish, mouth rinse) combined with toothpaste [25] and reported a D(M)FS pooled preventive fraction of 10% (95% CI, 2% to 17%; p = 0.01) in favour of a combined regimen over toothpaste alone but the significant difference in favour of the combined use of fluoride varnish and toothpaste accrued from a very small trial and appears likely to be a spurious result. The risks associated with these two interventions are small for the age group under investigation. A follow up study [26] of participants in the NW toothpaste trial [24] compared the prevalence of fluorosis in the study groups and reported a slight increase in prevalence of TF score 3 (an index of fluorosis) but no increase in the overall prevalence of developmental defects of enamel. Recent work shows that fluorosis risk is related to an elevated fluoride intake for all of the first 3 years of life [27] but that the first 2 years of life are the period with greatest risk [28].

Although sub-optimal, the available literature has informed the contents of Delivering Better Oral Health an Evidence Based Toolkit [29] national guidance that has been circulated by the Department of Health to every dental practice in England. The fluoride interventions to be investigated in this trial are identified by the Toolkit and the approach taken in this trial; focusing on caries free children is also supported by the Toolkit, which recommends application of the interventions to all children attending dental practice, the majority of whom will be caries free at their first attendance. This reasoning was informed by the outcomes of the North West cohort study [7] and because there are no effective screening tools to accurately and reliable identify the children who will develop caries. The effectiveness of the intervention to be tested and the impact on NHS costs are unknown and need to be tested in primary care.

The aim of this study is to therefore measure the costs and effects of a 'preventive package' of fluoride varnish, fluoride toothpaste, toothbrush and standard dental health education in keeping young children who regularly attend primary care service free of dental caries, compared with receiving standard dental health education alone.

If the technologies tested in this trial are effective at preventing caries and reducing costs it will influence how dentistry is provided for young children both in the UK and internationally. If the interventions are shown not to be an efficient use of resources this will also influence policy and commissioning, to perhaps focus prevention resources on population interventions such as water fluoridation.

## Methods/Design

### Trial Objectives

To compare over a 3 year period the effectiveness of fluoride varnish, fluoride toothpaste, toothbrush and standardised health education, provided twice a year, as a preventive package, with standardised health education alone provided twice a year in preventing the conversion of 2 to 3 year old children from caries-free at baseline to caries-active state in the primary dentition, reducing the number of carious surfaces (caries into dentine) in the primary dentition in children who convert from caries free to caries active states and preventing episodes of pain and extraction of primary teeth.

To compare over a 3 year period the costs of dental care in a group receiving fluoride varnish, fluoride toothpaste, toothbrush and standardised health education, provided twice a year as a preventive package with a group receiving standardised health education alone provided twice a year.

### Study design

**Figure 1:**
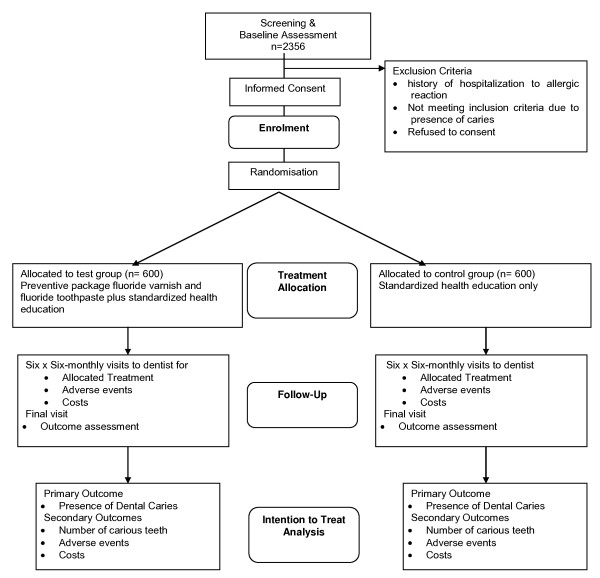
**Trial schematic showing the screening, recruitment and randomisation of children**.

NIC-PIP is a pragmatic three year parallel group randomised controlled trial where 2 and 3 year old children are randomised to receive the caries preventive package or not. The recruitment of the children is outlined in Figure 1. A full economic evaluation of the costs of the package is being conducted.

### Ethical considerations

Full ethical approval for this study has been obtained from Northwest 7 REC- GM Central (formally Central Manchester Research Ethics Committee). Two external bodies; a Data Monitoring and a Trial Steering Committee, will monitor study progress.

### Study Participants

Participants will be children who are initially aged 2 and 3.99 years who attend NHS General Dental Service (GDS) practices in Northern Ireland. Children will be eligible to participate in the study if they are aged 2 or 3 years, attending the selected GDS practices and a person with parental responsibility signs a consent form. The children will not be eligible if: they have caries into dentine, a past history of fillings or extractions due to caries, fissure sealants on primary molar teeth, history of severe allergic reactions requiring hospitalisation or are already participating in any other clinical trial involving medical products at recruitment.

### Trial interventions

The fluoride varnish (22,600 ppm) is classed as an Investigative Medicinal Product and therefore must comply with relevant UK regulations [30]. The fluoride varnish and toothpaste will appear in its normal commercial packaging. The fluoride varnish will be applied to the dried primary teeth of the children by a participating dentist at two visits to the dental surgery each year at approximately 6 monthly intervals (+/- 4 weeks). One drop of varnish will be applied to the primary teeth in each arch (2 drops in total) using a standardised brush applicator. After application parents will be advised not to brush their children's teeth for 24 hours.

A free toothbrush and a free 50 ml tube of 1,450 ppm of fluoride toothpaste will also be provided to test group children twice a year. Parents of children aged 2 but not 3 years will be advised to use a smear of toothpaste and those over 3 years will be advised to use a pea sized blob of toothpaste when brushing their teeth. Photographs of a smear and a pea size blob will be included in the standardised dental health education guide. It will be stressed to parents that children must be supervised by an adult when they brush their teeth. Standardised dental health education will be given at each 6 month check up visit by dentists following written guidance.

Children allocated to the control group will attend at 6 monthly intervals and receive the same standardised dental health education at each 6 month visit as the test group. The control group will not receive any professionally applied or provided fluoride interventions.

### Follow-up of children

**Figure 2:**
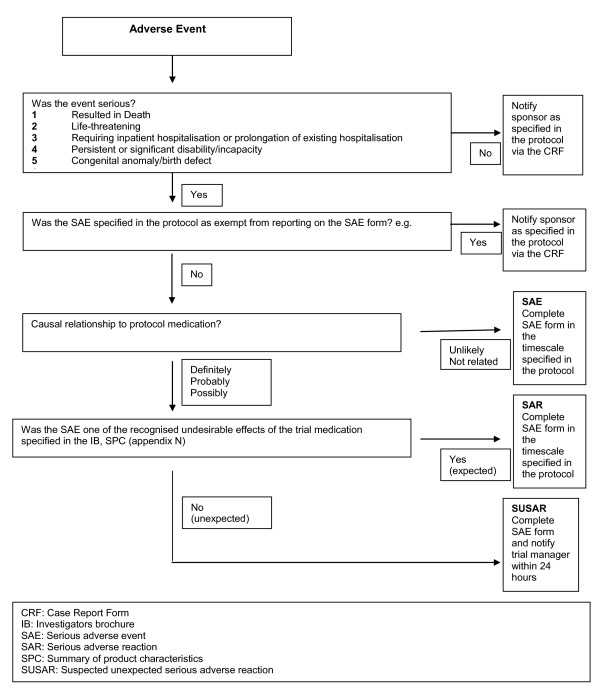
**Flow chart of assessing and notification of Adverse Events**.

Children will be followed from trial entry until the end of the study (three years). They will attend the dentist every 6 months where data on any symptoms reported or treatment received by each child at each visit (both planned 6 monthly and unplanned symptomatic visits) will be collected on site clinical record forms. A piloted questionnaire for the person with parental responsibility will be used to identify any unscheduled visits to other dental services and other episodes of toothache not severe enough to require a visit to the dentist. Fluoride varnish has been used routinely in dental practice and the risk to participants is low. The reporting criteria for adverse events is shown in Figure 2. The trial manager is undertaking duties delegated by the trial sponsor and is responsible for reporting of Suspected Unexpected Serious Adverse Reactions (SUSARs) and other Serious Adverse Reactions (SARs) to the competent authorities (regulatory authorities and central research ethics committees). The person with parental responsibility, and children, where appropriate, have the right to withdraw from the trial at any time for any reason, and this will be documented.

### Outcome measures

Baseline and outcome examinations at 3 years will be performed by trained and calibrated examiners. Outcome examiners will be blinded to the treatment allocation and using the same diagnostic protocol. The primary outcome is to measure whether the child has become caries active (caries into dentine). The following secondary outcomes are to be measured on the children: the number of carious surfaces (caries into dentine) in the primary dentition for children who have become caries active, the number of episodes of pain and number of extractions of primary teeth and the costs of dental care over a 3 year period. The final three outcomes will be assessed by questionnaire filled in by the person with parental responsibility.

### Randomisation

Potentially eligible children will be identified from the electronic databases of practices or in the case of practices without a computer by the Business Services Organisation. These children will be block-booked into dedicated trial sessions in the dental practices. A separate randomisation schedule will be prepared by the CRSC for each recruiting centre using randomised permutated blocks. The block lengths will vary to ensure the centres are blind to patient allocation. An invitation letter and trial information leaflet will be sent to parents of identified children asking if they would like to participate in the trial. An appointment for a check-up will be included in the invitation and will stress that the adult with parental responsibility for the child must accompany the child when they attend for assessment. The child's dentist or the external dental examiners (who will undertake the baseline examinations) will consent the children into the trial. Baseline assessment will be undertaken after each child has been consented but prior to randomisation.

An eligibility form will be faxed to the CRSC, where the children will be centrally randomised to one of the two treatment groups. The children will initially be identified at registration by their initials, date of birth and gender only. Once all of the eligibility criteria have been verified by the CTU they will provide the investigator with confirmation of the treatment allocation via fax to provide a paper record of the allocation, and the unique patient ID will be assigned.

### Sample size considerations

**Figure 3:**
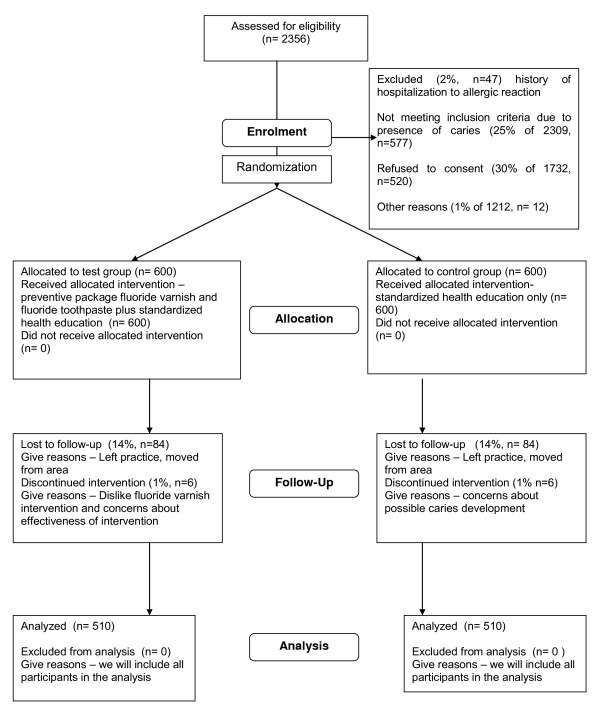
**CONSORT flowchart showing the estimated numbers of children throughout the trial**.

The principal outcome measure is whether the child develops caries in the primary dentition or not. We expect to see an absolute difference in the proportion of children with caries after 3 years of 0.1 between test and control groups. If caries free children are selected for the study it is estimated that 47% will develop caries over the three years. A two group chi-square test with a 0.050 two-sided significance level will have 90% power to detect the difference between a proportion of 0.470 and a proportion of 0.370 (odds ratio of 0.662) when the sample size in each group is 510. We assume that 2% will be excluded because of a history of severe allergic reaction and a further 1% for other reasons. We also assume that 75% of children approached will be caries free and a 70% consent rate with an estimated 15% drop-out rate over the 3 years. Therefore we will need to initially invite at least 2,356 children to take part in the study, recruiting 1200 children to ensure we have sufficient power at the end of the trial. The recruitment process is summarised in the CONSORT flow chart in Figure 3.

### Statistical analysis

All analyses will use an 'intention to treat' principal. All outcome measures stated in the protocol will be fully analysed using generalized linear models adjusting for covariates felt to be of prognostic importance including age and socio-economic status (SES). Statistical significance will be at the 0.05 level for all analysis and 95% confidence intervals will be calculated. A binary logistic regression model will be fitted to the primary outcome, whether the child remained caries free or not, with study group, age and socioeconomic status as covariates. The unadjusted and adjusted odds ratios from the logistic regression model will be reported and will specify that the adjusted is the primary analytical approach. A pre-planned subgroup analysis will be undertaken for deprived/not deprived children by selecting children whose parents are exempt from dental charges or not.

Health economic analysis will compare the total cost to the NHS for dental care in each of the two arms of the trial in accordance with the relative levels of effectiveness for each of the two arms. A multiple linear regression model will be fitted to the individual discounted costs per child with group, age and socio-economic status (SES) as covariates. If the assumptions underlying the model are not upheld, robust estimates of the standard errors will be calculated for the estimated parameters. Separate calculations will be made of between-treatment differences in cost to parents to identify any between-programme trade-offs. All calculations will be subjected to sensitivity analysis and discount rates of 3.5% for both cost and benefits will be applied. The incremental cost effectiveness ratio (ICER) will be estimated by dividing the difference in mean discounted costs between the two groups by the difference in discounted proportions that remain caries free. The relationship between cost and secondary outcomes such as the number of carious surfaces will be examined in an analogous fashion to that detailed above. The net present value of costs will be divided by the net present value of the number of carious surfaces to ascertain the average cost for carious surface avoided.

### Time plan for the NIC-PIP study

Patient recruitment began in April 2011 and is planned to continue until November 2011.

## Competing interests

The authors declare that they have no competing interests.

## Authors' contributions

MT, KMM and HVW were responsible for the initial research question, and contributing to drafting of the study protocol. MD, SK, CO, GC, MS, SN and MG have all also contributed to the development of the protocol and study design as members of the research team. HVW and MT were responsible for the drafting of this paper, although all authors provided comments on the drafts and have read and approved the final version.

## Pre-publication history

The pre-publication history for this paper can be accessed here:

http://www.biomedcentral.com/1472-6831/11/27/prepub
